# Perceptions of Frailty and Prehabilitation Among Thoracic Surgeons: Findings From a National Survey

**DOI:** 10.1016/j.atssr.2023.12.012

**Published:** 2024-01-20

**Authors:** Johnathan R. Kent, Julia Chavez, Daniel Rubin, Lauren J. Gleason, Andrea Landi, Megan Huisingh-Scheetz, Darren S. Bryan, Mark K. Ferguson, Jessica Donington, Maria Lucia Madariaga

**Affiliations:** 1 Department of Surgery, University of Chicago, Chicago, Illinois; 2 Department of Anesthesia and Critical Care, University of Chicago, Chicago, Illinois; 3 Department of Medicine, University of Chicago, Chicago, Illinois

## Abstract

**Background:**

Frailty is associated with increased perioperative morbidity and mortality. How thoracic surgeons recognize, measure, and mitigate frailty in their daily clinical practice is unknown. We administered a national survey to determine the current practices of thoracic surgeons managing frail patients.

**Methods:**

A 144-question survey developed in collaboration with the University of Chicago Survey Lab was sent to CTSnet.org members who identified as general thoracic surgeons, practiced in the United States, and had publicly available emails. Responses were collected from August 12 to September 11, 2022. Both fully and partially (at least 20%) completed surveys were included in a descriptive statistical analysis.

**Results:**

After 2796 surveys were administered, 342 surgeons responded. Surgeons were in practice a median of 23 years (range, 1-50 years) at academic (63.4% [187/295]) or community (36.6% [108/295]) centers. Most surgeons believed it important to assess frailty preoperatively (83.9% [287/342]), but only 28% (97/342) of surgeons performed routine frailty assessment. Barriers to routine frailty assessment included lack of tools (80.0% [32/40]), training (59.0% [23/39]), and staffing (56.4% [22/39]). Whereas most surgeons believed that frailty could be mitigated (72.2% [247/342]), only 49.5% (156/315) prescribed prehabilitation. Up to 78.7% (203/263) of surgeons would delay or cancel surgery for patient frailty, depending on disease cause.

**Conclusions:**

Thoracic surgeons recognize that frailty is an established risk factor for perioperative morbidity and mortality; however, there is high variability in diagnosis and management of frailty. Guidelines are needed to establish best practices for screening and mitigation to optimally treat frail patients.

## Visual Abstract

**Figure:**
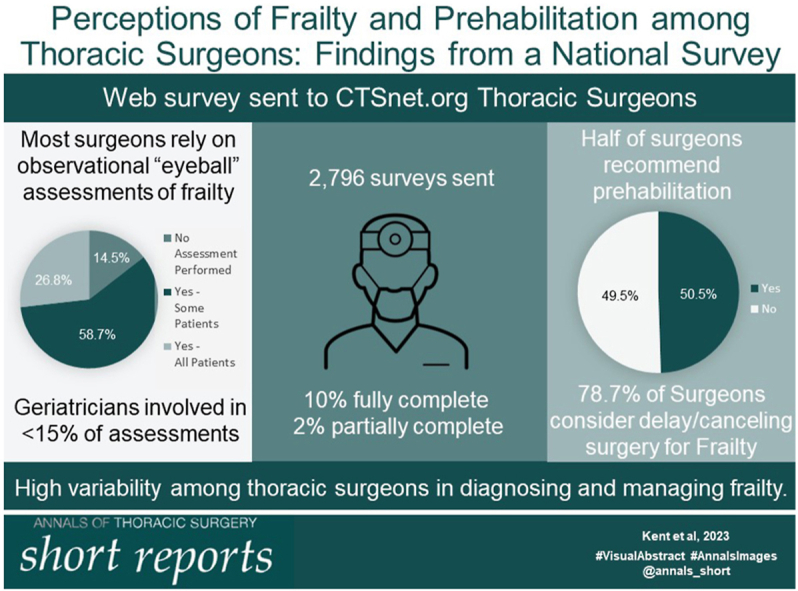

**In Short**

▪ Thoracic surgeons recognize frailty as an important factor affecting perioperative outcomes for their patients.
▪ Methods used by thoracic surgeons in the United States to screen for and to manage the frail patient are highly variable.

Frailty is an age-associated clinical syndrome characterized by limited physiologic reserve.^1^ Frailty is associated with increased risk of postoperative complications, length of stay, hospital cost, postdischarge institutionalization, unplanned readmission, and mortality.^2^, ^3^, ^4^, ^5^ Up to 12% of patients seen by thoracic surgeons are frail, with an additional 58% scoring as pre-frail.^6^ As the US population ages, the prevalence of frailty in thoracic surgery patients will continue to rise.

In 2021, The American Association for Thoracic Surgery released a consensus statement that identified frailty as an important factor in assessing risk for lung cancer resection.^5^ Despite this, how thoracic surgeons in the United States incorporate frailty screening and mitigation in their practice is unknown. In this study, we conducted a national survey to understand how thoracic surgeons recognize, measure, and mitigate frailty.

## Material and Methods

### Survey

To evaluate perceptions of frailty and prehabilitation, an anonymous web-based survey was distributed to CTSnet.org members who self-identified as general thoracic surgeons and practiced in the United States. The 144-question survey’s content underwent expert editing for best practice features in ordering, wording, and formatting by survey methodologists from the University of Chicago Survey Lab. The survey was beta tested by 4 thoracic surgeons in a qualitative review, and iterative revisions were made. This study was deemed exempt by the University of Chicago institutional review board (IRB22-1019; decision: November 9, 2022).

The final survey was administered with Qualtrics software (Appendix A). Questions were nested so that each respondent’s survey questions were prompted by the responses to prior queries, and no surgeon saw all 144 questions. Surgeons were emailed 3 times to encourage participation, and responses were collected between August and September 2022. Surveys that were <20% complete were considered incomplete and excluded from analysis.

### Statistical Analysis

All percentages are accompanied by the numbers from which they are derived to provide transparency of the sample size. Career stage of the surgeon was classified as early (0-7 years), mid (8-12 years), and late (>12 years). Surgeons who self-identified as working in either academic or university-affiliated environments were classified as academic, whereas all others were characterized as community. Comparisons between groups were made with *χ*^2^ tests for categorical variables and Student *t*-tests for continuous variables with statistical significance indicated by a *P* value < .05. Statistics were performed in R version 4.2.0 (R Foundation for Statistical Computing).

## Results

### Respondents

Of 2796 surveys administered, 286 (10%) were fully completed and 56 (2%) were partially completed for an American Association of Public Opinion Research Response Rate 4 of 11.2%.^7^ Responding surgeons were 90.3% male (261/289) and 80.1% white (225/281; Table 1). All respondents identified as attending surgeons, and 53.9% (159/295) reported working exclusively as general thoracic surgeons. Most surgeons (63.4% [187/295]) worked in academic environments, and most respondents were late-career surgeons (75.3% [216/287]).

**Table 1:** Demographic Characteristics of Respondents

Characteristic	Overall (N = 342)
Female	9.7 (28/289)
Race/ethnicity	
White	80.1 (225/281)
Asian	16.3 (46/281)
Hispanic	6.2 (18/291)
Black	3.2 (9/281)
Age, y	57.5 (28-82)
Years in practice	23 (1-50)
Practice (at least in part)	
Thoracic surgery	95.3 (281/295)
Cardiac surgery	38.3 (113/295)
Vascular surgery	12.2 (36/295)
Surgical oncology	5.76 (17/295)
General surgery	1.36 (4/295)
Thoracic transplantation	1.36 (4/295)
Intensivist	0.29 (1/295)
Perform thoracic surgery for ≥50% of practice	60 (177/295)
Practice environment	
Academic hospital	49.2 (145/295)
Community hospital	34.2 (101/295)
University-affiliated community	16.6 (49/295)
Private practice	5.42 (16/295)
Veterans Affairs hospital	5.08 (15/295)
Other	2.03 (6/295)

Categorical variables are presented as percentage (n/N). Continuous variables are presented as median (minimum-maximum).

### Perception of Frailty

Almost all surgeons reported being “very familiar” or “somewhat familiar” with frailty, with most (83.9% [287/342]) surgeons believing that frailty is “very important” to assess (Supplemental Table 1). Most surgeons believed that frailty had a “large” effect on both 30-day (69.3% [237/342]) and overall (88.6% [303/342]) operative outcomes. Almost all surgeons (81.4% [239/294]) were either “somewhat” or “strongly” in favor of the creation of societal guidelines for frailty screening and mitigation.

Surgeons were queried about patient demographic factors that could be associated with frailty. Characteristics that surgeons associated with increased frailty risk included smoking, cancer history, being underweight, and coming from a low-income environment (Supplemental Table 1).

### Frailty Screening Practices

Most surgeons performed frailty screening of selected patients by observation alone (58.7% [195/332]) or by using formal tools (32.5% [111/342]); few surgeons performed routine frailty assessment of all patients (Table 2). Surgeons most commonly believed that surgeon observation met the minimum threshold for frailty evaluation, followed by formal assessment and geriatrician evaluation (Supplemental Table 2). For those who did not perform frailty screening, the most common barriers to screening for frailty were lack of available tools (35% [14/40]), insufficient time or staffing (15.4% [6/39]), and need for additional training (12.8% [5/39]).

**Table 2:** Frailty Screening Practices

Do you record observational frailty assessments for patients ahead of a thoracic procedure (informal assessment)?	(N = 332)
No	48 (14.5)
Yes, some types of patients	195 (58.7)
Yes, all patients	89 (26.8)
When frailty is informally assessed, who does the observation?	(N = 280)
Surgeon	236 (84.3)
Geriatrician	12 (4.3)
Primary care physician	17 (6.1)
Other	31 (11.1)
Is frailty formally assessed using specific objective criteria from a chart review of frailty assessment tool for your patients?	(N = 342)
No	204 (59.6)
Yes, some types of patients	111 (32.5)
Yes, all patients	27 (7.9)
When frailty is formally assessed, who does the assessment?	(N = 138)
Surgeon	60 (43.5)
Geriatrician	22 (15.9)
Primary care physician	12 (8.7)
Other	48 (34.8)
How would you support or oppose establishment of societal guidelines for frailty screening and mitigation?	(N = 294)
Strongly oppose	10 (3.4)
Somewhat oppose	16 (5.4)
Neutral—no opinion	29 (9.9)
Somewhat favor	88 (30)
Strongly favor	151 (51.4)

Values are reported as number (percentage).

### Frailty Mitigation

Most surgeons believed that frailty could be mitigated (72.2% [247/342]; Supplemental Table 3). A majority (56.7% [178/314]) of surgeons believed that “most” or “virtually all” frail patients would benefit from prehabilitation (Figure). Close to half of respondents regularly prescribed prehabilitation to frail patients (49.5 % [156/315]), most often recommending optimizing nutrition, participating in mild to moderate exercise, or working with a physical therapist (Supplemental Figure).

**Figure:**
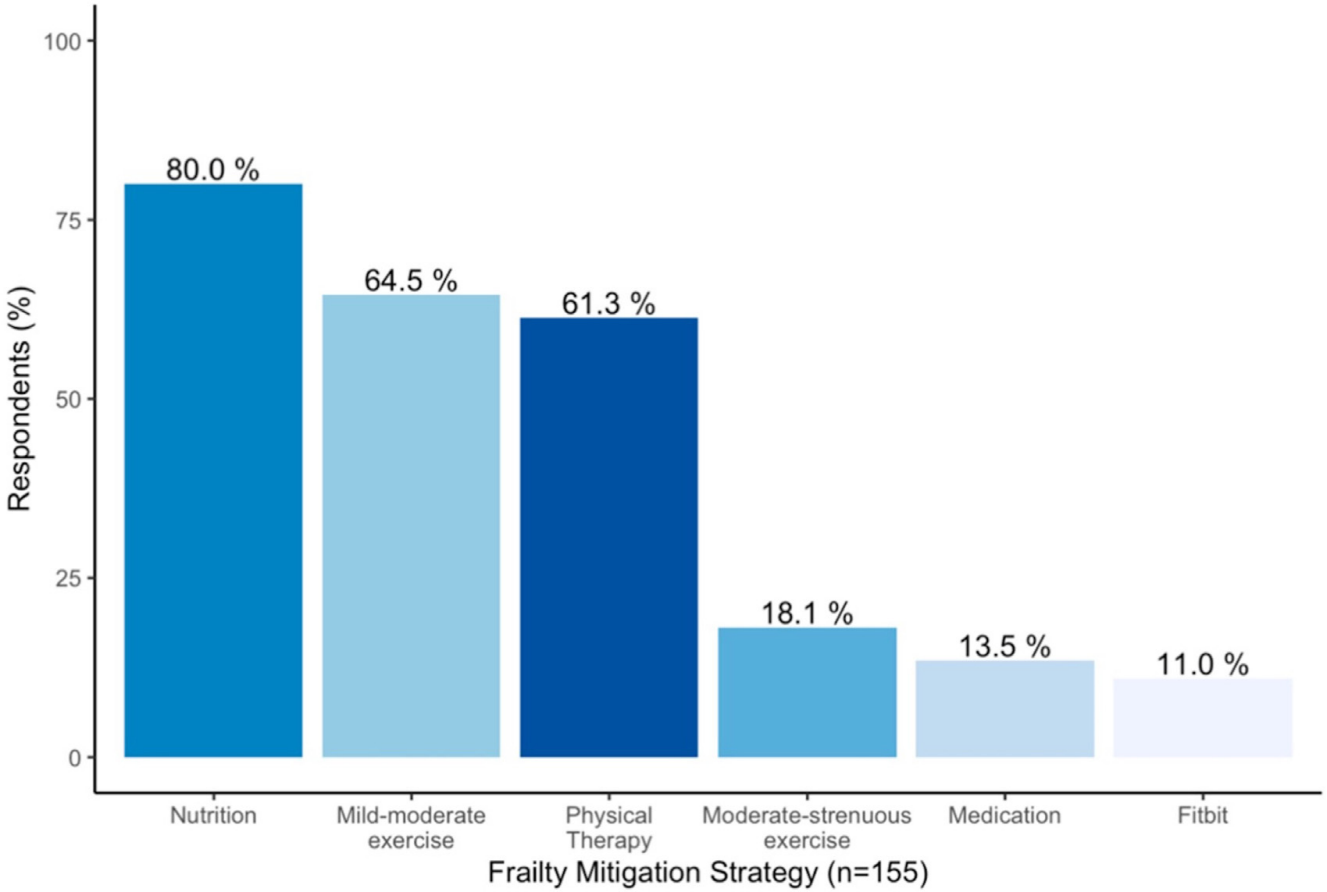
Frailty mitigation strategies used by thoracic surgeons.

Of the respondents who believed that prehabilitation benefits frail patients, most commonly believed that 3 to 5 weeks was the least amount of time needed for it to be effective before surgery (Supplemental Table 3).

### Impact of Frailty on Clinical Decision-Making

Surgeons typically assessed patients for frailty within 3 months of an operation for a benign condition and 1 month before an operation for a malignant condition (Supplemental Table 4). Most surgeons would be willing to delay surgery 3 to 5 weeks to allow prehabilitation for patients deemed frail (71.1% [216/304]). In situations in which prehabilitation failed to improve a patient’s physiologic status, most surgeons elected to cancel surgery (60.5% [101/167]). Surgeons were most likely to cancel or to delay surgery if the planned procedure was a pneumonectomy or an esophagectomy (Table 3).

**Table 3:** Impact of Frailty on Decision to Delay or to Cancel Surgery by Procedure Type

If a patient with the following conditions is assessed as being frail, how likely are you to delay or to cancel surgery?	Probably Not	Somewhat Likely	Very Likely
Wedge resection of solid nodule (n = 292)	48.6 (142)	37.0 (108)	14.4 (42)
Wedge resection of subsolid nodule (n = 291)	39.2 (114)	35.1 (102)	25.8 (75)
Segmentectomy or lobectomy for stage 1 NSCLC (n = 294)	30.3 (89)	48.3 (142)	21.4 (63)
Patient who is a candidate for SBRT (n = 260)	28.1 (73)	23.5 (61)	48.5 (126)
Esophagectomy for malignant esophageal disease (n = 263)	15.6 (41)	41.4 (109)	43.0 (113)
Pneumonectomy for stage 1 NSCLC (n = 282)	11.0 (31)	17.0 (48)	72.0 (203)
Esophagectomy for benign esophageal disease (n = 263)	4.2 (11)	17.1 (45)	78.7 (207)

Values are reported as percentage (number).

NSCLC, non-small cell lung cancer; SBRT, stereotactic body radiation therapy.

### Frailty-Specific Perioperative Care

Perioperative frailty programs were available at the institutions of 24.1% (71/295) of surgeons, but only 31.4% (22/70) found that these programs “greatly” improved outcomes. Goals of care discussions were performed as part of surgical planning process with all patients by 71.1% (216/304) of surgeons and with only some patients by 27.0% (82/304) of surgeons.

### Subgroup Analyses

Academic surgeons were less likely than community surgeons to routinely screen for frailty in all of their patients (22.9% [41/179] vs 35.5% [38/107]; *P* = .05) but were more likely than community surgeons to prescribe prehabilitation (55.6% [104/187] vs 40.7% [44/108]; *P* = .02). Compared with female surgeons, male surgeons were less likely to formally assess frailty (40.2% [105/261] vs 46.4% [13/28]; *P* = .02) and were more likely to perform frailty assessment by observation (86.5% [186/215] vs 65.2% [15/23]; *P* = .02). Younger surgeons (<50 years) trended toward being more likely to believe that frailty could be mitigated (82.5% [66/80] vs 70.7% [140/58]; *P* = .06).

## Comment

We evaluated perceptions of frailty and prehabilitation among surgeons performing general thoracic surgery in the United States. Most respondents reported at least some familiarity with frailty independent of practice setting and believed frailty assessment to be important. However, only a minority of respondents reported routinely screening all patients for frailty, and formal frailty assessments were infrequently performed. These findings suggest that whereas general thoracic surgeons recognize the importance of frailty screening and mitigation, there is a gap between knowledge and clinical practice.

Interestingly, whereas most thoracic surgeons report evaluating patients for frailty, the use of formal frailty assessment tools lags behind observational assessments. Informal, observational assessments of frailty (ie, the “eyeball” test) are particularly susceptible to observer bias, changing, for example, according to whether the observer is male or female.^8^ Provider bias in observational assessments may be compounded by lack of familiarity with patient factors associated with frailty.

Surgeons who are unaware that patients are more likely to be frail when female^9^ and non-Hispanic Black^10^ may be less likely to consider such patients frail during subjective evaluation by the eyeball test. Furthermore, we found that the impact of frailty on surgeons’ decision-making was dependent on procedure morbidity, with procedures associated with higher morbidity less likely to be offered to frail patients. Increased use of objective frailty assessment tools in a standardized manner could potentially limit the impact of observer bias on determination of frailty status and surgical candidacy.

Importantly, 81.4% of surgeons responding to our survey support establishment of societal guidelines for frailty screening and mitigation. In addition to providing a road map for assessing patients, a consensus statement may encourage surgeons to pursue a multidisciplinary approach to dealing with the medical complexities of the physiologically frail patient. Surgeons may benefit from geriatric expertise as they determine the duration, method, and efficacy of prehabilitation.

In our practice, we screen for frailty by the modified Fried frailty phenotype, which uses a combination of patient-provided subjective data (exhaustion, shrinkage, and activity level) with objective assessment (gait speed and grip strength), without relying on the patient’s comorbidity burden.^10^ We found that it is easily incorporated into clinic workflow because patients complete the survey portion in the waiting room and clinic staff administer physical assessments in less than 5 minutes while taking vital signs.^10^

### Limitations

This study has several limitations. The survey had a low response rate, included some partial respondents, and offered participation only to CTSnet.org members who practice in the United States, potentially limiting generalizability because of selection bias. In addition, whereas the study was intended to assess perceptions of general thoracic surgeons, 40% of respondents performed thoracic surgery as a minority of their practice, and our results may better capture the perceptions of cardiothoracic surgeons. Finally, the survey was self-reported, which may introduce recall bias in the responses. Despite these limitations, this publication provides a cross-sectional examination of current thoracic surgeon perceptions and practice in managing frailty.

### Conclusion

General thoracic surgeons recognize the importance of frailty but may not be consistently applying this knowledge in their clinical practice. Efforts to increase frailty screening and formal assessments of frailty through societal guidelines and inclusion of frailty metrics in national databases may be useful in changing surgeons’ practice. In addition, further research is needed to develop strategies to promote the consistent use of validated frailty assessment tools in clinical practice and to establish best practices for the prehabilitation of frail patients.
