# Supplementary material for: Perceptions of Frailty and Prehabilitation Among Thoracic Surgeons: Findings From a National Survey
Source: Ann Thorac Surg Short Rep. 2024 Jan 20;2(3):581–5. doi: 10.1016/j.atssr.2023.12.012 (PMC11708733; doi:10.1016/j.atssr.2023.12.012)
Supplement: Appendix [file mmc1.pdf]

Frailty Survey

- Material in blue print was not shown to respondents on screen.
- Variable names begin with “Q” except within square brackets [] that indicate questions for which each answer choice is a separate variable (grids and select all that apply).
- Codes corresponding to answer choices are in parentheses.
- Standard codes are used for Not Applicable (-4), Missing (-3), Don’t Know (-2) and Refused (-1). In this way, these responses can either be treated separately or as a group (< 0) in programs and analysis.
- Variables with __TEXT at the end of the variable name are text fields rather than codes with labels.

Start of Block: Block 1

QIntro
Thank you for your willingness to help us learn about your views and experiences related to patient frailty ahead of surgery.   The survey has 4 sections and takes about 10 minutes total. Would you like to go directly to the survey or learn more about this study?

- ○ Go directly to survey (1)
- ○ Learn more about the study (2)

Page Break

Display This Question:

If Thank you for your willingness to help us learn about your views and experiences related to patie... = Learn more about the study

What is this study about? This is a survey about frailty in thoracic surgery patients. This study is designed to learn more about the opinions and experiences of thoracic surgeons with respect to dealing with frail surgical patients. Results from this survey will be used to determine if efforts are needed to further educate thoracic surgeons about assessing frailty in the pre-operative setting.

Who is conducting this survey? Maria Lucia Madariaga, MD; Johnathan Kent, MD; Mark Ferguson, MD; Jessica Donington, MD; Julia Chavez; Ally Wang funded with Departmental Funds. Data collection is through Qualtrics, a survey platform, and managed by the University of Chicago Survey Lab.

Who is eligible to participate? US physicians who do thoracic surgery as a regular part of their practice and residents who are training in these areas are eligible to take part.

What will I experience if I take part? This is a web survey with four sections that, all together, takes about 10 minutes. There is no remuneration other than your satisfaction at contributing to better knowledge and practice, and no cost other than your time. The fours survey sections are: Your ideas about patient frailty, Assessing frailty, Managing Frailty in the Context of Surgery, Demographics

Who will know what I say? Your answers are confidential. Contact information is used by the University of Chicago Survey Lab solely to recruit participation. The Survey Lab deletes all contact information once data have been collected, and they send the PI and the data analysis team a dis-identified dataset with that information removed. In addition, your participation is completely voluntary. You may decline to answer any question you are not comfortable responding to, and may end your participation at any time.

What if I have additional questions or concerns? If you have any logistical problems taking the survey, please contact Kevin Ulrich, Hillary Hanson or Martha Van Haitsma at the University of Chicago Survey Lab:
Kevin Ulrich, Survey Lab Director: ulrichkv@uchicago.edu or 773-834-3667
Martha Van Haitsma, Survey Lab Co-Director: mvh@uchicago.edu or 773-834-3674
Hillary Hanson, Survey Lab Assistant Director: hansonhd@uchicago.edu

If you have questions about the research itself, please contact the principle investigator at: mlmadariaga@uchicago.edu

If you have concerns or questions about your rights as a study participant, please contact the University of Chicago Biological Sciences Division Institutional Review Board (BSDIRB) at 773-702-6505 and refer to study number IRB22-1019.

Page Break

Section 1 of 4: Your Ideas about Patient Frailty

Q1.1

How familiar are you with frailty as a clinical syndrome?

- ◯ Very familiar (4)
- ◯ Somewhat familiar (3)
- ◯ Have heard of it only (2)
- ◯ Prior to taking this survey I never heard of it (1)

Q1.2

How important do you believe it is to assess patient frailty ahead of thoracic surgery?

- ◯ Very important (4)
- ◯ Moderately important (3)
- ◯ A little important (2)
- ◯ Not important at all (1)

Page Break

Q1.3

Whether or not patients have formal frailty diagnoses, and *ex*cluding pre-frailty here as in all remaining survey questions, what proportion of your thoracic surgery clinic patients would you estimate are frail?

- ◯ 81 - 100% (12)
- ◯ 60 - 80% (11)
- ◯ 50 - 59% (10)
- ◯ 40 - 49% (9)
- ◯ 30 - 39% (8)
- ◯ 25 - 29% (7)
- ◯ 20 - 24% (6)
- ◯ 15 - 19% (5)
- ◯ 10 - 14% (4)
- ◯ 5 - 9% (3)
- ◯ 1 - 4% (2)
- ◯ Less than 1% (1)

Q1.4

How big an effect do you think frailty has on the *acute 30-day patient outcomes* of surgery?

- ◯ Large effect (4)
- ◯ Medium effect (3)
- ◯ Small effect (2)
- ◯ Non-significant (1)

Q1.5

How big an effect do you think frailty has on the length of time patients require to *completely recover* from surgery?

- ◯ Large effect (4)
- ◯ Medium effect (3)
- ◯ Small effect (2)
- ◯ Non-significant (1)

Q1.6

Do you think frailty can be mitigated at all prior to surgery?

- ◯ Yes (1)
- ◯ No (2)

Page Break

Display This Question: If Do you think frailty can be mitigated at all prior to surgery? DOES NOT EQUAL No

[Q1.7]

For the following types of frail patient, how much do you think frailty can *usually* be mitigated ahead of surgery?

	None at all (1)	A little bit (2)	Moderately (3)	A lot (4)
Q1.7A Those with very high levels of frailty this	◯	◯	◯	◯
Q1.7B Those with moderate levels of frailty	◯	◯	◯	◯
Q1.7C Those with marginal levels of frailty	◯	◯	◯	◯

Page Break

Display This Question: If Do you think frailty can be mitigated at all prior to surgery? DOES NOT EQUAL No

[Q1.8]

To mitigate frailty ahead of thoracic surgery, what would you be likely to recommend to a patient? Please select all that apply.

- ▢ **Q1.8A** Nutritional supplementation or other dietary recommendation
- ▢ **Q1.8B** Medication
- ▢ **Q1.8C** Mild to moderate general exercise that the patient does independently
- ▢ **Q1.8D** Moderate to more strenuous general exercise that the patient does independently
- ▢ **Q1.8E** Specific exercises for individual patients overseen by a physical therapist
- ▢ **Q1.8F** Prescribe a Fitbit or similar with a specific recommendation for increased exercise
- ▢ **Q1.8G** Other, please describe: **Q1.8G_TEXT** ________________________________
- ▢ **Q1.8H** I would not be likely to recommend anything to a patient ahead of surgery to mitigate frailty

End of Block: Block 1

Start of Block: Block 2

Section 2 of 4: Assessing Frailty

Q2.1

Do you routinely officially record the *observational* assessment you make about patient frailty ahead of a thoracic surgery procedure?

- ◯ Yes, all patients (1)
- ◯ Yes, some types of patients (2)
- ◯ No (3)
- ◯ N/A -- I do not routinely make observational assessments of patient frailty ahead of thoracic surgery procedures (-4)

Page Break

Q2.2

Beyond or in addition to observational assessment ahead of a thoracic surgical procedure, are patients in your clinic routinely *formally* assessed for frailty using specific objective criteria from a chart review or using a frailty-assessment tool?

- ◯ Yes, all patients (1)
- ◯ Yes, some types of patient (2)
- ◯ No (3)

Page Break

Display This Question:
If Do you routinely officially record the observational assessment you make about ... = Yes, all patients
Or Do you routinely officially record the observational assessment... = Yes, some types of patient

[Q2.3]

Who routinely does the *observational* assessment for frailty? Please select all those who routinely do this:

- ▢ **Q2.3A** The surgeon who will do the operation
- ▢ **Q2.3B** A consulting geriatrician
- ▢ **Q2.3C** The patient's primary care provider
- ▢ **Q2.3D** Somebody else, please describe their position: **Q2.3D_TEXT** __________

Display This Question: If Beyond or in addition to observational assessment ahead of a thoracic surgical procedure, are pat... = Yes, all patients Or if Beyond or in addition to observational assessment ahead of a thoracic surgical procedure, are pat... = Yes, some types of patient

[Q2.4]

Who routinely does the *formal* assessment for frailty? Please select all those who routinely do this:

- ▢ **Q2.4A** The surgeon who will do the operation
- ▢ **Q2.4B** A consulting geriatrician
- ▢ **Q2.4C** The patient's primary care provider
- ▢ **Q2.4D** Somebody else, please describe their position: **Q2.4D_TEXT** __________

Page Break

Display This Question If Do you routinely officially record the observational assessment you make ... = No

And if Beyond or in addition to observational assessment ahead of a thoracic surgical procedure, are pat... = No

[Q2.5]

Please note if any of the following are reasons why you do not routinely assess your patients for frailty.

	Main reason (1)	Contributing reason (2)	Not a reason (3)
Q2.5A You lack the tools to assess this.	◯	◯	◯
Q2.5B You lack the training to recognize when frailty assessment might be needed.	◯	◯	◯
Q2.5C This is (or should be) the responsibility of other providers or specialists.	◯	◯	◯
Q2.5D You lack time or staffing for this.	◯	◯	◯
Q2.5E This kind of assessment is not normally covered by insurance.	◯	◯	◯
Q2.5F This kind of assessment is not important for what you do - the results do not affect your decisions.	◯	◯	◯
Q2.5G Some other reason, please describe: Q2.5G_TEXT ________________________________	◯	◯	◯

Display This Question: If How important do you believe it is to assess patient frailty ahead of thoracic surgery? DOES NOT EQUAL Not important at all

[Q2.6]

Which of the following would you find *minimally adequate* for assessing patient frailty ahead of surgery?

	Enough by itself (1)	*Not* sufficient alone (2)	Can't say – *not* familiar with this (3)
Q2.6A Your own in-person patient observation.	◯	◯	◯
Q2.6B Frailty assessment tool that relies on chart review alone.	◯	◯	◯
Q2.6C Frailty assessment tool that requires current patient input (such as through questions or a survey) and physical testing (for example grip strength tests, walking tests or similar).	◯	◯	◯
Q2.6D Consult from a geriatrician.	◯	◯	◯
Q2.6E Primary Care Provider assessment.	◯	◯	◯

Page Break

Q2.7

Over the last full month that you worked, not counting weeks you were on vacation or sick leave, about how many different *new* clinic patients did you see?

▼ 0 (0) ... 100 or more (100)

Q2.8

Over the last full month that you worked, not counting weeks you were on vacation or sick leave, about how many different *return* clinic patients did you see?

▼ 0 (0) ... 100 or more (100)

Page Break

Display This Question: If Over the last full month that you worked, not counting weeks you were on vacation... DOES NOT EQUAL 0

Q2.9

About how many of those [SHOW N FROM Q2.7] *new* clinic patients you saw the last full month you worked were screened for frailty?

▼ 0 (0) ... 100 or more (100)

Display This Question: If Over the last full month that you worked, not counting weeks you were on vacation or sick leave,... DOES NOT EQUAL 0

Q2.10

About how many of those [SHOW N FROM Q2.8] *return* clinic patients were screened for *new-onset* frailty?

▼ 0 (0) ... 100 or more (100)

Page Break

Display This Question:
If Over the last full month that you worked, not counting weeks you were on vacation ... DOES NOT EQUAL 0
And About how many of those new clinic patients you saw the... DOES NOT EQUAL 0
And About how many of new clinic patients you saw the... , Is Displayed

Q2.11

Based on their screening results how many of those [SHOW N FROM Q2.9] *screened new* patients would you say are actually frail?

▼ 0 (0) ... 100 or more (100)

Display This Question:
If Over the last full month that you worked, not counting weeks you were on vacation... DOES NOT EQUAL 0
And About how many of those return clinic patients were scre... DOES NOT EQUAL 0
And About how many of those return clinic patients were scre... , Is Displayed

Q2.12

Based on their screening results, about how many of those [SHOW N FROM Q2.10] *screened-for-new-onset return* patients would you say are actually frail?

▼ 0 (0) ... 100 or more (100)

Page Break

[Q2.13]
Based on what you have read or experienced, all else equal, which patients are *more likely* to be frail …

Q2.13A Men or women?
- ◯ Men more likely to be frail (1)
- ◯ Both sexes equally likely to be frail (2)
- ◯ Women more likely to be frail (3)

Q2.14B Smokers or non-smokers?
- ◯ Smokers more likely to be frail (1)
- ◯ Both groups equally likely to be frail (2)
- ◯ Non-smokers more likely to be frail (3)

Q2.14C Cancer patients or patients with other conditions?
- ◯ Cancer patients more likely to be frail (1)
- ◯ Both groups equally likely to be frail (2)
- ◯ Non-cancer patients more likely to be frail (3)

Q2.14D Obese, normal weight or underweight patients?
- ◯ Obese most likely to be frail (1)
- ◯ Normal weight most likely to be frail (2)
- ◯ Underweight most likely to be frail (3)
- ◯ No differences in average frailty by weight (4)

Q2.14E Low, middle or high income patients?
- ◯ Low income most likely to be frail (1)
- ◯ Middle income most likely to be frail (2)
- ◯ High income most likely to be frail (3)
- ◯ No differences in average frailty by income level (4)

Q2.14F Low or high education patients?
- ◯ Less than college education more likely to be frail (1)
- ◯ Both groups equally likely to be frail (2)
- ◯ College or above education more likely to be frail (3)

Q2.14G White patients or specific groups of non-white patients?
- ◯ White patients are most likely to be frail (1)
- ◯ Black patients specifically are most likely to be frail (2)
- ◯ Hispanic patients specifically are most likely to be frail (3)
- ◯ Both Black patients and Hispanic patients are most likely to be frail (4)
- ◯ No differences in average frailty by race (5)

End of Block: Block 2

Start of Block: Block 3

Section 3 of 4: Managing Frailty in the Context of Surgery

Q3.1

Whether or not pre-habilitation is a practical possibility given healthcare resources, about what proportion of your *frail* patients do you think could medically benefit from pre-habilitation ahead of surgery?

- ◯ All or virtually all (5)
- ◯ Most (4)
- ◯ About half (3)
- ◯ Less than half (2)
- ◯ None or virtually none (1)

Display This Question: If Whether or not pre-habilitation is a practical possibility given healthcare resources... DOES NOT EQUAL None or virtually none

Q3.2

About what proportion of your frail patients have access to the kind of pre-habilitation ahead of surgery that you think they most need or would most benefit from?

- ◯ All or virtually all (5)
- ◯ Most (4)
- ◯ About half (3)
- ◯ Less than half (2)
- ◯ None or virtually none (1)

Page Break

Q3.3

Do you or your practice prescribe pre-habilitation?

- ◯ Yes (1)
- ◯ No (2)

Display This Question: If Do you or your practice prescribe pre-habilitation? = Yes

[Q3.4]

What type of prehab do you prescribe? Please select all that apply.

- ▢ **Q3.4A** Nutritional supplementation or other dietary recommendation
- ▢ **Q3.4B** Medication
- ▢ **Q3.4C** Mild to moderate general exercise that the patient does independently
- ▢ **Q3.4D** Moderate to more strenuous general exercise that the patient does independently
- ▢ **Q3.4E** Specific exercises for individual patients overseen by a physical therapist
- ▢ **Q3.4F** Prescribe a Fitbit or similar with a specific recommendation for increased exercise
- ▢ **Q3.4G** Something else, please describe: **Q3.4G_TEXT** ____________________

Page Break

Q3.5

How often do you refer your frail patients to a geriatrician or refer them for comprehensive geriatric assessment?

- ◯ Always or virtually always (5)
- ◯ Most of the time (4)
- ◯ About half the time (3)
- ◯ Sometimes (2)
- ◯ Hardly ever or never (1)

Page Break

Display This Question: If Do you think frailty can be mitigated at all prior to surgery? DOES NOT EQUAL No

Q3.6

What is the *least* amount of time needed for prehab– the time below which pre-habilitation is unlikely to confer any benefit?

- ◯ Any amount of pre-hab can help (0)
- ◯ 1 week (1)
- ◯ 2 weeks (2)
- ◯ 3 weeks (3)
- ◯ 4 weeks (4)
- ◯ 5 weeks (5)
- ◯ 6 or more weeks (6)

Display This Question: If Do you think frailty can be mitigated at all prior to surgery? DOES NOT EQUAL No

Q3.7

What is the *most* amount of time prehab is needed – the time past which pre-habilitation is unlikely to noticeably improve surgical outcomes?

- ◯ 6 weeks or less (6)
- ◯ 7 weeks (7)
- ◯ 8 weeks (8)
- ◯ 9 weeks (9)
- ◯ 10 weeks (10)
- ◯ 11 weeks (11)
- ◯ 12 or more weeks (12)

Page Break

Q3.8

For a benign condition, how many weeks on average are typically available between a diagnosis of frailty and a scheduled surgery?

- ◯ More than 3 months ahead of surgery (1)
- ◯ Within 3 months of surgery (2)
- ◯ Within 1 month of surgery (3)
- ◯ Within 2 weeks of surgery (4)
- ◯ Assess frailty too rarely to report a time frame as typical (5)

Q3.9

For a cancerous condition, how many weeks on average are typically available between a diagnosis of frailty and a scheduled surgery?

- ◯ More than 3 months ahead of surgery (1)
- ◯ Within 3 months of surgery (2)
- ◯ Within 1 month of surgery (3)
- ◯ Within 2 weeks of surgery (4)
- ◯ Assess frailty too rarely to report a time frame as typical (5)

Q3.10

If a frail patient has cancer, *on average* how long are you willing to hold off on surgery for prehab?

- ◯ No time (0)
- ◯ 1 week (1)
- ◯ 2 weeks (2)
- ◯ 3 weeks (3)
- ◯ 4 weeks (4)
- ◯ 5 weeks (5)
- ◯ 6 weeks (6)
- ◯ 7 weeks (7)
- ◯ 8 weeks (8)
- ◯ More than 8 weeks (9)

Page Break

Q3.11

Have you ever delayed surgery for pre-hab?

- ◯ Yes (1)
- ◯ No (2)

Display This Question: If Have you ever delayed surgery for pre-hab? = Yes

Q3.12

Has prehab ever failed to improve a patient?

- ◯ Yes (1)
- ◯ No (2)

Display This Question: If Has prehab ever failed to improve a patient? = Yes

[Q3.13]

What are all the ways you have responded to a patient in pre-hab failing to improve ahead of surgery? Select all those you have experience with.

- ▢ **Q3.13A** Went ahead with the surgery as this seemed more urgent
- ▢ **Q3.13B** Postponed the surgery further
- ▢ **Q3.13C** Canceled the surgery
- ▢ **Q3.13D** Something else, please describe: **Q3.13D_TEXT** __________________

Page Break

Q3.14

Before establishing a care plan, do you routinely talk with patients about their care goals related to disability/dependence and quality of life vs. cure or life-expectancy?

- ◯ Yes, with all my patients (1)
- ◯ With some patients (2)
- ◯ No (3)

Display This Question: If Before establishing a care plan, do you routinely talk with ... = With some patients

Q3.15

Is frailty a condition that triggers having this discussion in advance of establishing a care plan?

- ◯ Yes, standard with all frail patients (1)
- ◯ Sometimes -- not with all frail patients (2)
- ◯ No (3)

Page Break

[Q3.16]

If a patient with the following conditions is assessed as being frail, how likely are you to delay or cancel surgery?

	Very likely to cancel or delay (3)	Somewhat likely to cancel or delay (2)	Probably not cancel or delay (1)	N/A – Not an operation I do (-4)
**Q3.16A** Solid lung nodule requiring a wedge resection	○	○	○	○
**Q3.16B** Subsolid lung nodule requiring a wedge resection	○	○	○	○
**Q3.16C** Stage I lung cancer requiring a segment/lobectomy	○	○	○	○
**Q3.16D** Stage I lung cancer requiring a pneumonectomy	○	○	○	○
**Q3.16E** SBRT is a good alternative for this case	○	○	○	○
**Q3.16F** Benign condition requiring an esophagectomy	○	○	○	○
**Q3.16G** Cancerous condition requiring an esophagectomy	○	○	○	○

Q3.17

If your patient is a smoker at the time you determine they will need surgery, do you delay surgery until they quit?

- ○ Usually -- this is the default (5)
- ○ More than half the time (4)
- ○ About half the time (3)
- ○ Sometimes (2)
- ○ Never or hardly ever -- *not* delaying for this is the default (1)

Q3.18

If a patient is assessed as being frail, how would this be likely to affect your choice of open thoracotomy vs. minimally invasive thoracic surgery?

- ○ Would push me *strongly* toward minimally invasive (1)
- ○ Would push me *somewhat* toward minimally invasive (2)
- ○ *Not* likely to affect my decision (3)

Q3.19

Does your hospital offer specialized peri- or post-operative care programs for frail or pre-frail surgical patients?

- ○ Yes (1)
- ○ No (2)

Display This Question: If Does your hospital offer specialized peri- or post-operative care ... = Yes

Q3.20 What has been the effect of this specialized care program on the quality of care for frail or pre-frail patients?

- ◯ Greatly improved it (4)
- ◯ Somewhat improved it (3)
- ◯ Had no effect (2)
- ◯ Reduced the quality of care (1)
- ◯ No point of comparison to say – the program was in place when I got here (-1)

Display This Question: If Does your hospital offer specialized peri- or post-operative care ... = No

Q3.21

Given what you know about your hospital structure and any past quality improvement programs, what would you predict the effect of a specialized care program for frail or pre-frail patients would be on the quality of their care?

- ◯ Greatly improve (4)
- ◯ Somewhat improve (3)
- ◯ Have no effect (2)
- ◯ Reduce the quality of care (1)

Page Break

Q3.22

How strongly would you favor or oppose establishment of medical society guidelines for frailty screening and mitigation in the context of surgery?

- ◯ Strongly favor (5)
- ◯ Somewhat favor (4)
- ◯ Neutral - no opinion (3)
- ◯ Somewhat oppose (2)
- ◯ Strongly oppose (1)

Q3.23_TEXT

If you have further comments or ideas about frailty to share before completing the demographic questions, please add those here.

____

____

Please continue to next page

End of Block: Block 3

Start of Block: Block 4

Section 4 of 4: Demographics

Please help us understand our results by sharing the following. Recall that all information is confidential and not linked to any individuals.

Q4.1

Are you a …

- ◯ Resident (1)
- ◯ Fellow (2)
- ◯ Attending physician (3)
- ◯ Other, please specify (4) **Q4.1_TEXT** ____

[Q4.2]

Which of the following are part of your routine surgical caseload? Select all that apply.

- ☐ **Q4.2A** General surgery
- ☐ **Q4.2B** Surgical oncology
- ☐ **Q4.2C** Thoracic surgery
- ☐ **Q4.2D** Cardiac surgery
- ☐ **Q4.2E** Vascular surgery
- ☐ **Q4.2F** Other specialized surgical areas, please list: **Q4.2F_TEXT** _____________

Page Break

Display This Question: If Which of the following are part of your routine surgical caseload? SELECTED COUNT FOR Q4.2 Is Greater Than 1

[Q4.3]

Roughly what proportion of your surgeries fall into each area? Proportions should sum to 100.

Display This Choice: If Which of the following are part of your routine surgical caseload? = General surgery

Q4.3A General: ________ [PERCENT]

Display This Choice: If Which of the following are part of your routine surgical caseload? = Surgical oncology

Q4.3B Surgical Oncology: ________ [PERCENT]

Display This Choice: If Which of the following are part of your routine surgical caseload? = Thoracic surgery

Q4.3C Thoracic: ________ [PERCENT]

Display This Choice: If Which of the following are part of your routine surgical caseload? = Cardiac surgery

Q4.3D Cardiac: ________ [PERCENT]

Display This Choice: If Which of the following are part of your routine surgical caseload? = Vascular surgery

Q4.3E Vascular: ________ [PERCENT]

Display This Choice: If Which of the following are part of your routine surgical caseload? = Other specialized surgical areas And Which of the following are part of your surgical work? Text Response Is Not Empty

Q4.3F [INPUT TEXT FIELD FROM Q4.2 OTHER SPECIFY]: ________ [PERCENT]

Display This Choice: If Which of the following are part of your routine surgical caseload? = Other specialized surgical areas And Which of the following are part of your surgical work? Text Response Is Empty

Q4.3G Other type of surgery: ________ [PERCENT]

Total: _________ (Total displayed to Respondent – should add to 100)

Page Break

Q4.4
About how many years have you been in practice?

	0 5 10 15 20 25 30 35 40 45 50
Approximate years ()	

[Q4.5]
What best characterizes your current practice environment? Select all that apply.

- ▢ **Q4.5A** Academic teaching hospital
- ▢ **Q4.5B** University-affiliated community hospital
- ▢ **Q4.5C** Community hospital
- ▢ **Q4.5D** VA or military hospital
- ▢ **Q4.5E** Private practice
- ▢ **Q4.5F** Something else, please describe: **Q4.5F_TEXT** ________________________

Q4.6_TEXT
What is the zip code of the hospital where you most practice?

Hospital zip code: ____

Page Break

Display This Question:

If If What is the zip code of the hospital where you most practice? Text Response Is Empty

Q4.7
In what state is the hospital where you (most) practice located?

▼ Alabama (1) ... Puerto Rico (52)

Display This Question: If What is the zip code of the hospital where you most practice? Text Response Is Empty

Q4.8_TEXT
In what city or town is the hospital where you (most) practice located?

____

Page Break

Q4.9
What is your gender identity?

- ◯ Male (1)
- ◯ Female (2)
- ◯ Non-binary (3)
- ◯ A different identity, please specify: (4) **Q4.9_TEXT** ________________________________

Q4.10
What year were you born?

▼ 2001 (2001) ... 1940 or earlier (1940)

Q4.11

Do you identify as Latino/a (Latinx), Hispanic or of Spanish Origin?

- ◯ Yes (1)
- ◯ No (2)

Page Break

[Q4.12]

Please select *all* racial categories that are part of your identity:

- ▢ **Q4.12A** East Asian (Chinese, Taiwanese, Japanese, Korean or other East Asian)
- ▢ **Q4.12B** Southeast Asian (Vietnamese, Cambodian, Laotian, Indonesian, Maylaysian, Thai or other Southeast Asian)
- ▢ **Q4.12C** South Asian (Indian, Pakistani, Bangladeshi, Sri Lankan, Nepalese or other South Asian)
- ▢ **Q4.12D** Black / African-American / Afro-Caribbean / Black African
- ▢ **Q4.12E** Middle-Eastern / North African / Arab-American
- ▢ **Q4.12F** Native American / Alaskan Native/ Indigenous person / American Indian
- ▢ **Q4.12G** Native Hawaiian or Pacific Islander
- ▢ **Q4.12H** White / Caucasian / European-American
- ▢ **Q4.12I** Other, please specify: **Q4.12I_TEXT** ________________________________

Page Break

Those are all the questions we have.
***Thank You* for your help with this study!**
We greatly appreciate your time and input.

End of Block: Block 4
