# Supplementary material for: Perceptions of Frailty and Prehabilitation Among Thoracic Surgeons: Findings From a National Survey
Source: Ann Thorac Surg Short Rep. 2024 Jan 20;2(3):581–5. doi: 10.1016/j.atssr.2023.12.012 (PMC11708733; doi:10.1016/j.atssr.2023.12.012)
Supplement: Supplementary Tables [file mmc2.docx]

**Supplemental Table/Figure Legends**

## Supplemental Table 1. Perceptions of frailty as a marker of peri-operative risk and its relationship to associated demographic factors.

Supplemental Table 2. Perceptions on adequacy of various methods for assessing frailty.

Supplemental Table 3. Frailty mitigation and prehabilitation.

Supplemental Figure 1. Frailty mitigation strategies used by thoracic surgeons.

Supplemental Table 4. Frailty & Surgical Practice.

## Supplemental Table 1.

|  | Overall (N=342)  n (%) | Academic (N=187)  n (%) | Community (N=108)  n (%) | P value |
| --- | --- | --- | --- | --- |
| **How familiar are you with frailty as a clinical syndrome?** | N = 342 | N=187 | N=108 |  |
| Prior to this survey I never heard of it | 8 (2.3%) | 3 (1.6%) | 1 (0.9%) | 0.235 |
| Have heard of it only | 17 (4.97%) | 9 (4.8%) | 5 (4.6%) |  |
| Somewhat familiar | 160 (46.8%) | 99 (52.9%) | 45 (41.7%) |  |
| Very familiar | 157 (45.9%) | 76 (40.6%) | 57 (52.8%) |  |
| **Is it important to assess for frailty prior to thoracic surgery?** | N = 342 | N= 187 | N=108 |  |
| A little important | 6 (1.75%) | 5 (2.67%) | 0 (0%) | 0.228 |
| Moderately important | 49 (14.3%) | 26 (13.9%) | 16 (14.8%) |  |
| Very important | 287 (83.9%) | 156 (83.4%) | 92 (85.2%) |  |
| **Which patients are more likely to be frail, …** |  |  |  |  |
| **Men or women** | N = 321 | N= 187 | N=108 |  |
| Both sexes equally likely to be frail | 186 (57.9%) | 122 (65.2%) | 50 (46.3%) | <0.001 |
| Women more likely to be frail | 107 (33.3%) | 43 (23.0%) | 53 (49.1%) |  |
| Men more likely to be frail | 28 (8.7%) | 22 (11.8%) | 5 (4.63%) |  |
| **Smokers or non-smokers** | N = 321 | N = 187 | N= 108 |  |
| Both groups equally likely to be frail | 69 (21.5%) | 42 (22.5%) | 22 (20.4%) | 0.519 |
| Smokers more likely to be frail | 248 (77.3%) | 144 (77.0%) | 84 (77.8%) |  |
| Non-smokers more likely to be frail | 4 (1.2%) | 1 (0.535%) | 2 (1.85%) |  |
| **Cancer patients or patients with other** **conditions** | N = 321 | N = 187 | N= 108 |  |
| Both groups equally likely to be frail | 86 (26.8%) | 46 (24.6%) | 31 (28.7%) | 0.3 |
| Cancer patients more likely to be frail | 234 (72.9%) | 141 (75.4%) | 76 (70.4%) |  |
| Non-cancer patients more likely to be frail | 1 (0.3%) | 0 (0%) | 1 (0.926%) |  |
| **Obese, normal weight or underweight** **patients** | N = 321 | N = 187 | N= 108 |  |
| No differences in average frailty by weight | 59 (18.4%) | 38 (20.3%) | 14 (13.0%) | 0.395 |
| Underweight most likely to be frail | 206 (64.2%) | 119 (63.6%) | 72 (66.7%) |  |
| Normal weight most likely to be frail | 2 (0.6%) | 1 (0.535%) | 1 (0.926%) |  |
| Obese most likely to be frail | 54 (16.8%) | 29 (15.5%) | 21 (19.4%) |  |
| **Low-, middle- or high-income patients** | N = 321 | N = 187 | N= 108 |  |
| No differences in average frailty by income level | 132 (41.1%) | 74 (39.6%) | 49 (45.4%) | 0.425 |
| Low income most likely to be frail | 182 (56.7%) | 110 (58.8%) | 55 (50.9%) |  |
| Middle income most likely to be frail | 3 (0.9%) | 1 (0.535%) | 2 (1.85%) |  |
| High income most likely to be frail | 4 (1.2%) | 2 (1.07%) | 2 (1.85%) |  |
| **Low or high education patients** | N= 320 | N = 187 | N= 108 |  |
| Both groups equally likely to be frail | 176 (55%) | 102 (54.8%) | 62 (57.4%) | 0.848 |
| Less than college education more likely to be frail | 137 (42.8%) | 79 (42.5%) | 44 (40.7%) |  |
| College or above education more likely to be frail | 7 (2.2%) | 5 (2.69%) | 2 (1.85%) |  |
| **White patients or specific groups of non-white patients** | N = 320 | N = 187 | N= 108 |  |
| No differences in average frailty by race | 234 (73.1%) | 131 (70.4%) | 84 (77.8%) | 0.049 |
| Black patients specifically are most likely to be frail | 18 (5.6%) | 14 (7.53%) | 3 (2.78%) |  |
| Both Black patients and Hispanic patients are most likely to be frail | 39 (12.2%) | 28 (15.1%) | 8 (7.41%) |  |
| Hispanic patients specifically are most likely to be frail | 1 (0.3%) | 1 (0.538%) | 0 (0%) |  |
| White patients are most likely to be frail | 28 (8.8%) | 12 (6.45%) | 13 (12.0%) |  |
| **What effect do you believe frailty has on your patients 30-day recovery?** | N = 342 | N = 187 | N= 108 |  |
| Small effect | 10 (2.92%) | 6 (3.21%) | 2 (1.85%) | 0.785 |
| Medium effect | 95 (27.8%) | 52 (27.8%) | 30 (27.8%) |  |
| Large effect | 237 (69.3%) | 129 (69.0%) | 76 (70.4%) |  |
| **What effect do you believe frailty has on your patients’ overall outcomes?** | N = 342 | N = 187 | N= 108 |  |
| Small effect | 2 (0.59%) | 2 (1.07%) | 0 (0%) | 0.229 |
| Medium effect | 37 (10.8%) | 25 (13.4%) | 9 (8.33%) |  |
| Large effect | 303 (88.6%) | 160 (85.6%) | 99 (91.7%) |  |

Supplemental Table 2.

| Which of the following is minimally adequate when assessing a patient for frailty pre-operatively? | Enough by itself  % (n) | Not sufficient alone  % (n) | Can’t say  Not familiar  % (n) |
| --- | --- | --- | --- |
| Surgeon in person observation (n=332) | 46.4 (154) | 50.6 (168) | 3.0 (10) |
| Frailty assessment tool that relies on chart review (n=331) | 7.9 (26) | 73.4 (243) | 18.7 (62) |
| Frailty assessment that requires observation and physical testing (n=332) | 38.3 (127) | 41.9 (139) | 19.9 (66) |
| Consult from a geriatrician (n=333) | 21.3 (71) | 46.5 (155) | 32.1 (107) |
| Primary Care Provider Assessment (n=332) | 8.1 (27) | 75.6 (251) | 16.3 (54) |

Supplemental Table 3.

|  | n (%) |
| --- | --- |
| **Do you believe that frailty can be mitigated pre-operatively?** | N = 342 |
| No | 95 (27.8%) |
| Yes | 247 (72.2%) |
| **If you believe that frailty can be mitigated…** |  |
| **How much can “Very High” frailty be mitigated?** | N = 247 |
| None at all | 30 (12.1%) |
| A little bit | 157 (63.6%) |
| Moderately | 50 (20.2%) |
| A lot | 10 (4%) |
| **How much can “Moderate” frailty be mitigated?** | N = 247 |
| None at all | 0 (0%) |
| A little bit | 51 (20.6%) |
| Moderately | 161 (65.2%) |
| A lot | 35 (14.2%) |
| **How much can “Marginal” frailty be mitigated?** | N = 247 |
| None at all | 1 (0.4%) |
| A little bit | 37 (15%) |
| Moderately | 92 (37.2%) |
| A lot | 117 (47.4%) |
| **For a frail patient would you recommend:** | N = 247 |
| Nutritional optimization | 225 (91.1%) |
| Mild-moderate exercise | 188 (76.1%) |
| Working with a physical therapist | 167 (67.6%) |
| Moderate-strenuous exercise | 44 (17.8%) |
| Other | 23 (9.3%) |
| Medication | 43 (17.4%) |
| Prescribe a fitbit | 48 (19.4%) |
| **What proportion of frail patients do you think would benefit from prehabilitation?** | N = 314 |
| None or virtually none | 22 (7%) |
| Less than half | 59 (18.8%) |
| About half | 55 (17.5%) |
| Most | 91 (29%) |
| All or virtually all | 87 (27.7%) |
| **What proportion of frail patients do you think have access to prehabilitation?** | N = 293 |
| None or virtually none | 80 (27.3%) |
| Less than half | 120 (41%) |
| About half | 37 (12.6%) |
| Most | 44 (15%) |
| All or virtually all | 12 (4.1%) |
| **Do you prescribe prehabilitation?** | N = 315 |
| No | 159 (50.5%) |
| Yes | 156 (49.5%) |
| **If so, what do you prescribe?** | N = 155 |
| Nutritional optimization | 124 (80%) |
| Mild-moderate exercise | 100 (64.5%) |
| Working with a physical therapist | 95 (61.3%) |
| Moderate-strenuous exercise | 28 (18.1%) |
| Other | 27 (17.4%) |
| Medication | 21 (13.5%) |
| Prescribe a fitbit | 17 (11%) |
| **How often does determining a patient to be frail result in a referral to a geriatrician?** | N = 313 |
| Hardly ever or never | 236 (75.4%) |
| Sometimes | 49 (15.7%) |
| About half the time | 6 (1.9%) |
| Most of the time | 17 (5.4%) |
| Always or virtually always | 5 (1.6%) |
| **What is the least amount of time for prehabilitation to be effective?** | N = 229 |
| 1-2 weeks | 63 (27.5%) |
| 3-5 weeks | 102 (44.5%) |
| 6 or more weeks | 25 (10.9%) |
| Any amount of prehabilitation can help | 39 (17%) |
| **What is the most amount of time for prehabilitation to be effective?** | N = 229 |
| 6 weeks or less | 76 (33.2%) |
| 7-8 weeks | 83 (36.2%) |
| 9-11 weeks | 19 (8.3%) |
| 12 or more weeks | 51 (22.3%) |

Supplemental Table 4.

|  | Overall (N=342)  n (%) | Academic (N=187)  n (%) | Community (N=108)  n (%) | P value | |
| --- | --- | --- | --- | --- | --- |
| **Typically, how much time prior to surgery is a patient evaluated for frailty…** |  |  |  |  | |
| **For a benign condition?** | N = 304 | N = 187 | N = 107 | |  |
| Within 2 weeks of surgery | 23 (7.6%) | 10 (5.35%) | 11 (10.3%) | | 0.17 |
| Within 1 month of surgery | 100 (32.9%) | 59 (31.6%) | 39 (36.4%) | |  |
| Within 3 months of surgery | 114 (37.5%) | 71 (38.0%) | 40 (37.4%) | |  |
| More than 3 months ahead of surgery | 38 (12.5%) | 29 (15.5%) | 8 (7.48%) | |  |
| Assess frailty too rarely to report a time frame as typical | 29 (9.5%%) | 18 (9.63%) | 9 (8.41%) | |  |
| **For a malignant condition?** | N = 303 | N = 186 | N = 107 | |  |
| Within 2 weeks of surgery | 64 (21.1%) | 35 (18.8%) | 26 (24.3%) | | 0.389 |
| Within 1 month of surgery | 190 (62.7%) | 125 (67.2%) | 62 (57.9%) | |  |
| Within 3 months of surgery | 25 (8.3%) | 15 (8.06%) | 9 (8.41%) | |  |
| More than 3 months ahead of surgery | 0 (0%) | 0 (0%) | 0 (0%) | |  |
| Assess frailty too rarely to report a time frame as typical | 24 (7.9%) | 11 (5.91%) | 10 (9.35%) | |  |
| **How long would you be willing to delay a cancer surgery for prehabilitation?** | N = 304 | N = 186 | N = 108 | |  |
| 1-2 weeks | 41 (13.5%) | 22 (11.8%) | 17 (15.7%) | | 0.276 |
| 3-5 weeks | 175 (57.6%) | 116 (62.4%) | 54 (50.0%) | |  |
| 6-8 weeks | 69 (22.7%) | 40 (21.5%) | 28 (25.9%) | |  |
| More than 8 weeks | 5 (1.6%) | 2 (1.08%) | 2 (1.85%) | |  |
| No time | 14 (4.6%) | 6 (3.23%) | 7 (6.48%) | |  |
| **Have you ever delayed surgery for prehabilitation?** | N = 304 | N = 187 | N = 108 | |  |
| No | 88 (28.9%) | 50 (26.7%) | 34 (31.5%) | | 0.462 |
| Yes | 216 (71.1%) | 137 (73.3%) | 74 (68.5%) | |  |
| **If you have delayed surgery for prehabilitation, has it ever failed to improve a patient?** | N = 216 | N = 107 | N = 74 | |  |
| No | 49 (22.7%) | 32 (23.4%) | 17 (23.0%) | | 1 |
| Yes | 167 (77.3%) | 105 (76.6%) | 57 (77.0%) | |  |
| **If prehabilitation has failed to improve a patient, have you...** | N = 167 | N = 105 | N = 57 | |  |
| **Canceled the surgery?** |  |  |  | |  |
| No | 66 (39.5%) | 49 (46.7%) | 17 (29.8%) | | 0.0554 |
| Yes | 101 (60.5%) | 56 (53.3%) | 40 (70.2%) | |  |
| **Proceeded with surgery?** |  |  |  | |  |
| No | 79 (47.3%) | 47 (44.8%) | 29 (50.9%) | | 0.562 |
| Yes | 88 (52.7%) | 58 (55.2%) | 28 (49.1%) | |  |
| **Postponed the surgery?** |  |  |  | |  |
| No | 88 (52.7%) | 53 (50.5%) | 34 (59.6%) | | 0.34 |
| Yes | 79 (47.3%) | 52 (49.5%) | 23 (40.4%) | |  |
| **Do you have goals of care conversations with your patients pre-operatively?** | N = 304 | N = 187 | N = 108 | |  |
| No | 6 (2%) | 4 (2.14%) | 1 (0.926%) | | 0.588 |
| With some patients | 82 (27%) | 48 (25.7%) | 32 (29.6%) | |  |
| Yes, with all my patients | 216 (71.1%) | 135 (72.2%) | 75 (69.4%) | |  |
| **If you only have goals of care conversations with some patients, does frailty trigger these discussions?** | N = 82 | N = 48 | N = 32 | |  |
| No | 2 (2.64 | 2 (4.17%) | 0 (0%) | | 0.483 |
| Sometimes -- not with all frail patients | 36 (43.9%) | 20 (41.7%) | 15 (46.9%) | |  |
| Yes, standard with all frail patients | 44 (53.7%) | 26 (54.2%) | 17 (53.1%) | |  |
| **Do you delay surgery if a patient is actively smoking?** | N = 294 | N = 186 | N = 107 | |  |
| Never or hardly ever -- not delaying for this is the default | 114 (38.8%) | 67 (36.0%) | 46 (43.0%) | | 0.529 |
| Sometimes | 77 (26.2%) | 48 (25.8%) | 29 (27.1%) | |  |
| About half the time | 13 (4.4%) | 10 (5.38%) | 3 (2.80%) | |  |
| More than half the time | 13 (4.4%) | 10 (5.38%) | 3 (2.80%) | |  |
| Usually -- this is the default | 77 (26.2%) | 51 (27.4%) | 26 (24.3%) | |  |
| **Does a diagnosis of frailty make you more likely to pursue a minimally invasive approach?** | N = 294 | N = 186 | N = 107 | |  |
| Not likely to affect my decision | 95 (32.3%) | 63 (33.9%) | 32 (29.9%) | | 0.783 |
| Would push me somewhat toward minimally invasive | 42 (14.3%) | 26 (14.0%) | 16 (15.0%) | |  |
| Would push me strongly toward minimally invasive | 157 (53.4%) | 97 (52.2%) | 59 (55.1%) | |  |
| **Do you have a perioperative program for frail patients at your hospital?** | N = 295 | N = 186 | N = 108 | |  |
| No | 224 (75.9%) | 134 (72.0%) | 89 (82.4%) | | 0.0628 |
| Yes | 71 (24.1%) | 52 (28.0%) | 19 (17.6%) | |  |
| **What effect does your hospitals perioperative program for frailty have on patients?** | N = 65 | N = 47 | N= 18 | |  |
| Had no effect | 4 (6.2%) | 3 (5.88%) | 1 (5.26%) | | 0.935 |
| Somewhat improved it | 39 (60%) | 29 (56.9%) | 10 (52.6%) | |  |
| Greatly improved it | 22 (33.8%) | 15 (29.4%) | 7 (36.8%) | |  |
| **What effect would you predict a perioperative program for frailty would have on your patients if your hospital had one?** | N = 224 | N = 134 | N = 89 | |  |
| Have no effect | 17 (7.6%) | 10 (7.46%) | 7 (7.87%) | | 0.576 |
| Somewhat improve | 137 (61.2%) | 79 (59.0%) | 58 (65.2%) | |  |
| Greatly improve | 70 (31.3%) | 45 (33.6%) | 24 (27.0%) | |  |
